# Prostate Cancer Incidence Rates in Africa

**DOI:** 10.1155/2011/947870

**Published:** 2011-08-01

**Authors:** Lisa W. Chu, Jamie Ritchey, Susan S. Devesa, Sabah M. Quraishi, Hongmei Zhang, Ann W. Hsing

**Affiliations:** ^1^Division of Cancer Epidemiology and Genetics, National Cancer Institute, National Institutes of Health, U.S. Department of Health and Human Services, Bethesda, MD 20852-7234, USA; ^2^Department of Epidemiology and Biostatistics, Arnold School of Public Health, University of South Carolina, Columbia, SC 29208, USA

## Abstract

African American men have among the highest prostate cancer incidence rates in the world yet rates among their African counterparts are unclear. In this paper, we compared reported rates among black men of Sub-Saharan African descent using data from the International Agency for Research on Cancer (IARC) and the National Cancer Institute Surveillance, Epidemiology, and End Results Program for 1973–2007. Although population-based data in Africa are quite limited, the available data from IARC showed that rates among blacks were highest in the East (10.7–38.1 per 100,000 man-years, age-adjusted world standard) and lowest in the West (4.7–19.8). These rates were considerably lower than those of 80.0–195.3 observed among African Americans. Rates in Africa increased over time (1987–2002) and have been comparable to those for distant stage in African Americans. These patterns are likely due to differences between African and African American men in medical care access, screening, registry quality, genetic diversity, and Westernization. Incidence rates in Africa will likely continue to rise with improving economies and increasing Westernization, warranting the need for more high-quality population-based registration to monitor cancer incidence in Africa.

## 1. Introduction

African American men have among the highest reported prostate cancer rates in the world [1, 2]. However, whether similarly high rates occur among men in Africa is unclear [3]. Previous reports from Africa were mostly limited to case series and hospital-based data, largely due to the difficulty in establishing high-quality population-based cancer registries in Africa [3–6]. Because West Africans and African Americans share a common genetic ancestry yet have very different lifestyles, a better understanding of prostate cancer rates and patterns among Sub-Saharan Africans may provide unique insights into the etiology of this disease [7]. Therefore, we examined available 1973–2007 incidence rates from Sub-Saharan Africa and the United States (US). 

## 2. Materials and Methods

We used prostate cancer incidence data for Africa from publications of the International Agency for Research on Cancer (IARC; http://www-dep.iarc.fr/): (1) Cancer Incidence in Five Continents (CI5), volumes IV-IX [4, 8] and (2) Cancer in Africa: Epidemiology and Prevention [3]. We included only registries that reported at least 10 cases of prostate cancer that were diagnosed during each time period and from countries that had populations that were more than 95% Black African or reported rates specific to Blacks. Twelve African registries fit these criteria (see Table 1); none of the registries in North or Central Africa met the inclusion criteria. Of the 12 registries selected, nine registries are population based with data collected at the national (The Gambia and Swaziland) or regional (Conakry, Guinea; Bamako, Mali; Niamey, Niger; Ibadan, Nigeria; Eldoret, Kenya; Blantyre, Malawi; Kyadondo, Uganda; Harare, Zimbabwe: African) levels. Of the other three registries, Blantyre, Malawi did not have cancer information based on death certificates due to absence of death registration in the country, South Africa is primarily pathology-based, and Namibia is primarily pathology based with some cases registered from the oncology services in its capital and largest city of Windhoek; it was not reported whether South Africa or Namibia included cancer information from death certificates.

For comparison with these African data, we calculated rates for US Blacks and Whites from the National Cancer Institute (NCI) Surveillance, Epidemiology, and End Results (SEER) Program for the original nine registries combined using SEER*Stat version 7.0.4 (http://www.seer.cancer.gov/ seerstat; NCI, Bethesda, Md) for total prostate cancer [10]. Although SEER expanded to include 13 registries in 1992 and further to 17 registries in 2000, to maintain geographic homogeneity over time, we restricted our analysis to the original 9 registries throughout. SEER annually provides updated rates, which for the earlier years are very similar to those reported in CI5 (data not shown). SEER includes stage-specific data, which are not reported in CI5. Classification of the prostate cancer stage at diagnosis has not been consistent over the 35-year period of the SEER program, with difficulties becoming apparent in delineating localized versus regional stage and more recently localized versus unknown stage. It appeared, however, that the definition and determination of distant-stage disease was more consistent over time, so we calculated rates according to SEER historical stage distant versus nondistant, including localized/regional and stage unknown [10]. The SEER November 2004 submission data file was used to calculate the stage-specific rates for cases diagnosed during 1973–1987 [11] and the November 2009 data file for cases diagnosed during 1988–2007 [10]. Cases that are distant stage at diagnosis in the US are likely to be clinically apparent and perhaps more comparable to most cases diagnosed in Africa. All rates were directly age adjusted to Segi's world standard population [9] and expressed per 100,000 man-years. 95% confidence intervals (CI) extracted from CI5, were estimated for data from Cancer in Africa (see table footnote for method), or provided by SEER*Stat. 

We examined trends for SEER during 1973–2007 and for three African registries that reported rates for at least three time points during that time period. We used log scales to plot the rates such that a slope of 10 degrees portrayed a change of 1% per year (Origin version 8.0; OriginLab Corporation, Northampton, Ma, USA) [12]. 

## 3. Results and Discussion

Among the African countries, the number of cases ranged from 20 in The Gambia (1997-1998) to 3,432 in South African Blacks (1989–1992) (Table 1). Incidence varied substantially by region, with rates highest in the East (10.7–38.1 per 100,000 man-years), intermediate in the South (14.3–21.8), and lowest in the West (4.7–19.8). The reported rate for Harare, Zimbabwe (38.1 during 1998–2002) was 8 times the rate in The Gambia (4.7 during 1997-1998). In comparison, rates among US Blacks were considerably higher up to 40 times those in Africa: 195.3 in US Blacks during 1993–1997 versus 4.7 in The Gambia during 1997-1998.

Reasons for the large variation of prostate cancer in blacks within the African continent and the observed East-West disparity are unclear but are likely related to differences in medical care access, registry quality, including completeness of case ascertainment and estimates of populations at risk, screening practices, as well as lifestyle factors in subpopulations [4]. For most of Africa, medical care access is limited, with only 4% of Ghanaian men in 2004–2006, for instance, having health insurance (unpublished data); in contrast, about 80% of non-Hispanic blacks in the U.S. had some type of health insurance coverage in 2008 [13]. In the more developed country of South Africa, diagnostic and screening facilities may be more accessible to the general population, but the racial disparity seen in prostate cancer incidence between blacks and whites [7] suggest that blacks may still have poorer access to medical care. Postapartheid, access to medical aid for whites were about seven times that of blacks [14]. Underdiagnosis of prostate cancer is likely in populations with limited health care access [3, 7]. 

Quality of the medical care systems and registries also may have a substantial impact on the completeness and accuracy of the reported incidence rates. Availability of pathology services (reflected by percent of cases microscopically verified; Table 1) likely compromises the quality of cancer diagnosis. For example, in The Gambia, which had the lowest prostate cancer incidence rate, only 20% of cases were morphologically verified during 1997-1998 [4]. In contrast, in Harare, Zimbabwe, which had the highest incidence rate, 63% of cases had morphological verification during 1998–2002 [8]. Both countries had much lower pathological confirmation rates of cancer than the US, where more than 93% of cases have been histologically confirmed since 1973 [10]. On the other hand, a high confirmation rate, such as in Namibia (97%) and South African Blacks (100%), suggests that the registry relied primarily on pathology records and that nonconfirmed cases were not included. A high proportion of cases that were ascertained based on death certificates only suggests that case finding has failed to identify cases that have not died, again potentially resulting in rate underestimation. This may occur in populations with limited infrastructure to support comprehensive data collection [3, 15], especially when diseases like cancer are less of a priority [16, 17]. Thus, the true prostate cancer incidence in African men is likely higher than what is reported here. There also may be uncertainties in the accuracy of the population enumerations and estimates of person-years at risk [4, 8], which could result in either under- or overestimation of the rates. 

**Figure 1:**
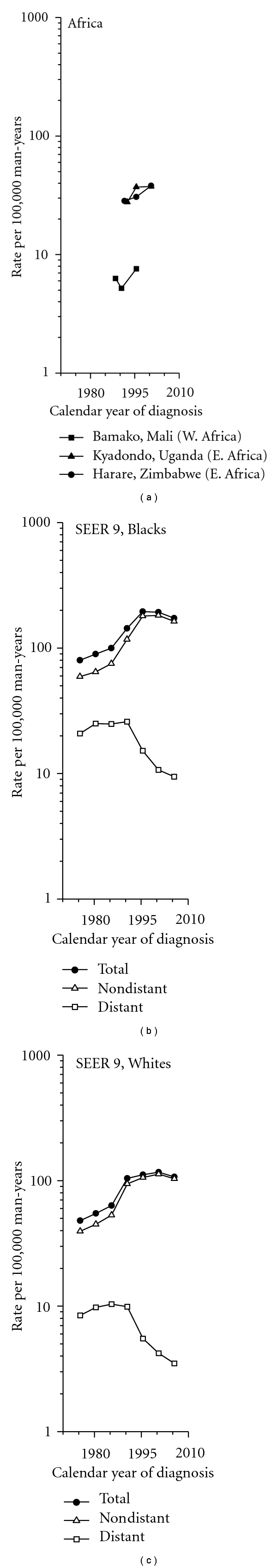
Age-adjusted (Segi's world standard) prostate cancer incidence in Sub-Saharan Africa and the United States, 1973–2007. (a) Africa: total prostate cancer rates from registries in three African cities; the populations of both Mali and Uganda were >95% black, and the rates for Zimbabwe were specific for black Africans. US: SEER nine registries combined for blacks (b) and whites (c): total and by SEER historical stage: nondistant and distant. All rates are for 3–5 year time periods (see Table 1).

Unlike the US where increasing and widespread use of prostate-specific antigen (PSA) screening contributed to the rapid rise in incidence during the early 1990s [18], the rising Sub-Saharan African rates were similar to the increases seen for total rates in the US before PSA screening was implemented (Figure 1). PSA screening is still uncommon in most parts of Sub-Saharan Africa, with reported prevalence of 2.5% in Ghana (unpublished data) and 4% in Senegal [19]. Within the SEER data, rates were consistently higher among blacks than whites, rose through the 1990s, especially rapidly during 1980s–1990s overall and for nondistant disease stage before leveling off during the 2000s, and declined notably for distant stage since 1990. Notably, while the total prostate cancer rates in the US were consistently much higher than those in Africa, total rates in East Africa (Uganda and Zimbabwe) were similar to the distant-stage rate among black Americans during the 1980s. Total rates in East Africa have also been higher than distant-stage rates reported for black and white Americans in recent years. This observation is also consistent with the fact that screening is uncommon in Africa, and thus cancers are more likely diagnosed at a more advanced stage. In fact, advanced disease accounted for 75% of cases in Ghana (unpublished data) and 47.9% in Senegal [20].

Similar to reasons given above for the geographic variation in rates within Africa, it is likely that improved health care systems and better ascertainment and reporting of cases may contribute to the rising rates in Africa [7, 21]. However, it is also possible that increased westernization in Africa in recent years, including changes in diet and lifestyle, may also play a role. For example, recent population-based data from Ghana show that the prevalence of obesity, a potential effect of Westernization, increased from 5% in 1998 [22] to 9% in 2004–2006, and the prevalence of overweight increased from 17% to 32% (unpublished data). US non-Hispanic Black men had a prevalence of obesity and overweight of 34.0% and 69.1%, respectively, in 2003-2004 [23]. Both clinical and etiologic investigations in African men are needed to further clarify reasons for the rising prostate cancer incidence in Africa.

Considering that the level of Westernization in Africa is still much lower than that in the US, the observation that total incidence rates in East Africa (Zimbabwe and Uganda), even in the earlier time period, were slightly higher than those of distant stage disease among African Americans is consistent with recent findings from genome-wide association studies (GWAS) showing that genetics are an important factor in prostate cancer. Recent GWAS have linked over 30 independent genetic loci to higher risks of prostate cancer in populations of European descent, including multiple loci in chromosome 8q24 [24–35]. Notably, some of the known risk alleles in 8q24 are more common in African Americans than non-African populations [28], suggesting that genetic variation may contribute to racial disparities between African American and other populations. In a large study of GWAS-identified risk variants and prostate cancer in African Americans, significant associations were found for some of the GWAS-identified risk variants in the same direction and of similar magnitude as those reported in men of European descent [36]. Most notably, all reported risk loci at 8q24 were significantly associated with prostate cancer with 8q24 region 2 attaining genome-wide significance levels. A recent GWAS specific to men of African descent also found similar results for previously identified variants in 8q24 but discovered an additional susceptibility locus at 17q21 [37]. It is noteworthy that the frequency of the 17q21 risk variation (rs7210100) is 4 to 7% in men of African ancestry, including Ghanaian men (7%), but is less than 1% in non-African populations (based on data from the 1000 Genomes Project). This novel finding suggests that some risk loci may be specific to African populations. Whether 8q24, 17q21, or other risk variants play an important role in prostate cancer in African men warrants further confirmation, and future studies are needed to determine their underlying biological mechanisms.

In a previous publication, Parkin et al. [7] found that the highest estimated rates of prostate cancer in Africa were seen in the South followed by Central, West, East, and North African regions. However, these 2008 estimates were for regional populations of all races combined and thus are not necessarily specific to blacks. For example, Parkin et al. [7] noted that the high rate of 40.5 per 100,000 man-years reported for Southern Africa was the composite of the rates among various racial groups. In South Africa alone, the rates ranged from a high of 41.1 per 100,000 man-years among whites, to 25.4 among mixed races, 14.3 among blacks, and 13.0 among Indians [3]. Because race is a well-established risk factor for prostate cancer, a more comparable assessment of prostate cancer rates in Africa for comparison with African Americans necessitates comparison of black-specific rates, as in the current study. 

## 4. Conclusions

Although data are limited, our analysis showed that (1) reported total prostate cancer incidence in Africa is lower than that among African Americans; (2) rates vary substantially (8-fold) within Sub-Saharan Africa, with rates lowest in the West and highest in the East; (3) total prostate cancer rates in Africa are similar to distant-stage disease rates in the US; (4) incidence appears to be rising in several African countries. It should be noted that when making inferences from these findings, consideration should be given to limitations in data quality. Undoubtedly, with improved economies and clinical diagnosis as well as increased Westernization, incidence rates in Africa are likely to continue to rise. Therefore, a high priority in this population should be the implementation of high-quality population-based cancer registration to monitor incidence rates in Africa and to develop effective cancer prevention strategies. 

## Figures and Tables

**Table 1:** Age-adjusted prostate cancer incidence rates^a^ per 100,000 man-years, 95% confidence intervals (CIs), percent microscopically verified, and percent reported by death certificate only in Sub-Saharan Africa and the United States, 1973–2007.

Location and/or race	Source	Time period	No. cases	Incidence rate^a^	95% CI^b^	Microscopically verified (%)	Death certificate only (%)
*East Africa *							
Blantyre, Malawi	Cancer in Africa	2000-2001	30	10.7	6.9–14.5	47	NK
Eldoret, Kenya	Cancer in Africa	1998–2000	54	16.8	12.3–21.3	30	NK
Harare, Zimbabwe: African	CI5 VII	1990–1992	112	28.3	22.5–43.1	64	9
	CI5 VIII	1993–1997	251	30.7	26.5–34.9	56	15
	CI5 IX	1998–2002	418	38.1	34.1–42.1	63	15
Kyadondo, Uganda	CI5 VII	1991–1993	86	27.7	21.6–33.8	67	NK
	CI5 VIII	1993–1997	215	37.1	31.7–42.5	77	0
	CI5 IX	1998–2002	262	37.6	32.8–42.4	58	NK

*Southern Africa *							
Namibia	Cancer in Africa	1995–1998	352	21.8	19.5–24.1	97	NK
South Africa: blacks	Cancer in Africa	1989–1992	3432	14.3	13.8–14.8	100	NK
Swaziland	Cancer in Africa	1996–1999	153	21.5	18.1–24.9	24	NK

*West Africa *							
Bamako, Mali	CI5 VI	1987–1989	21	6.3	3.5–9.1	5	5
	CI5 VII	1988–1992	33	5.2	3.4–7.0	21	6
	CI5 VIII	1994–1996	29	7.6	4.8–10.4	55	3
Conakry, Guinea	Cancer in Africa	1996–1999	62	9.7	7.3–12.1	45	NK
Ibadan, Nigeria	Cancer in Africa	1998–1999	115	19.8	16.2–23.4	70	NK
Niamey, Niger	Cancer in Africa	1993–1999	41	10.8	7.5–14.1	34	NK
The Gambia	CI5 VIII	1997-1998	20	4.7	2.5–6.9	20	NK

*North America *							
United States							
Blacks	NCI-SEER	1973–1977	2666	80	77.0–83.1	93	1
	NCI-SEER	1978–1982	3783	89.8	86.8–92.6	95	1
	NCI-SEER	1983–1987	4754	100.0	97.1–102.8	96	1
	NCI-SEER	1988–1992	7511	143.3	140.1–146.6	97	0
	NCI-SEER	1993–1997	10853	195.9	191.6–199.1	96	1
	NCI-SEER	1998–2002	11940	192.9	186.6–193.7	97	1
	NCI-SEER	2003–2007	12618	172.8	169.8–176.0	98	1

Whites	NCI-SEER	1973–1977	24212	47.9	47.3–48.5	94	1
	NCI-SEER	1978–1982	31389	54.8	54.1–55.3	95	1
	NCI-SEER	1983–1987	39492	63.5	62.8–64.0	97	0
	NCI-SEER	1988–1992	68863	104.3	103.3–104.9	96	1
	NCI-SEER	1993–1997	73687	111.8	110.5–112.2	97	1
	NCI-SEER	1998–2002	80100	116.9	115.1–116.8	97	1
	NCI-SEER	2003–2007	80022	107.0	106.2–107.8	98	1

CI: confidence interval; NK: not known; CI5: Cancer Incidence in Five Continents; NCI-SEER: National Cancer Institute's Surveillance, Epidemiology, and End Results Program: nine registries.

^a^All rates are age adjusted to Segi's world standard population [9]; African rates are shown only for populations at least 95% black or are specific for black Africans.

^b^95% CIs were obtained directly from CI5, were estimated for data from the Cancer in Africa publication by multiplying the standard error (incidence rate divided by the square root of the total number of cases) by 1.96, and adding to and subtracting from the incidence rate to obtain the upper and lower bounds, respectively, or were provided by SEER*Stat.
